# Probiotic assessment and antioxidant characterization of *Lactobacillus plantarum* GXL94 isolated from fermented chili

**DOI:** 10.3389/fmicb.2022.997940

**Published:** 2022-11-17

**Authors:** Yingjun Zhou, Wenbing Gong, Chao Xu, Zuohua Zhu, Yuande Peng, Chunliang Xie

**Affiliations:** Institute of Bast Fiber Crops, Chinese Academy of Agricultural Sciences, Changsha, China

**Keywords:** lactic acid bacteria, *Lactobacillus plantarum*, antioxidant activity, probiotic, hydrogen peroxide

## Abstract

Oxidative stress is caused by an imbalance between prooxidants and antioxidants, which is the cause of various chronic human diseases. Lactic acid bacteria (LAB) have been considered as an effective antioxidant to alleviate oxidative stress in the host. To obtain bacterium resources with good antioxidant properties, in the present study, 113 LAB strains were isolated from 24 spontaneously fermented chili samples and screened by tolerance to hydrogen peroxide (H_2_O_2_). Among them, *Lactobacillus plantarum* GXL94 showed the best antioxidant characteristics and the *in vitro* antioxidant activities of this strain was evaluated extensively. The results showed that *L. plantarum* GXL94 can tolerate hydrogen peroxide up to 22 mM, and it could normally grow in MRS with 5 mM H_2_O_2_. Its fermentate (fermented supernatant, intact cell and cell-free extract) also had strong reducing capacities and various free radical scavenging capacities. Meanwhile, eight antioxidant-related genes were found to up-regulate with varying degrees under H_2_O_2_ challenge. Furthermore, we evaluated the probiotic properties by using *in vitro* assessment. It was showed that GXL94 could maintain a high survival rate at pH 2.5% or 2% bile salt or 8.0% NaCl, live through simulated gastrointestinal tract (GIT) to colonizing the GIT of host, and also show higher abilities of auto-aggregation and hydrophobicity. Additionally, the usual antibiotic susceptible profile and non-hemolytic activity indicated the safety of the strain. In conclusion, this study demonstrated that *L. plantarum* GXL94 could be a potential probiotic candidate for producing functional foods with antioxidant properties.

## Introduction

Probiotics are defined as “live microorganisms which when administered in adequate amounts, confer health benefits to the host” (Hill et al., 2014). The majority of probiotics belong to the lactic acid bacteria (LAB) group. Lactic acid bacteria (LAB) are a diverse group of gram-positive bacteria that widely exist in nature including plants, animals and fermented foods, which was generally recognized as safe (GRAS) microorganisms, have a wide range of industrial applications, such as food fermentation, pharmaceutical, chemical and other industries (Feng and Wang, 2020). *Lactobacillus*, *Bifidobacteria*, and *Streptococcus* are the most famous genera that are described (Kiani et al., 2021). *Lactobacillus* is a fundamental group of LAB and is generally regard as safe. It is also the most common LAB strains in fermented foods (Das et al., 2016). The therapeutic effect of *Lactobacillus* fermented milk on gastrointestinal diseases has been found for a long time, and increasing experimental evidences in recent years have also made human understanding of *Lactobacillus* to a new height (Kok and Hutkins, 2018). Numerous studies have shown that some *Lactobacillus* strains benefit the host by improving balancing intestinal microbiota (Schnupf et al., 2018; He et al., 2020), regulating immunity (Galdeano and Perdigón, 2006; Håkansson et al., 2019), reducing cholesterol (Ebel et al., 2014; Fuentes et al., 2016), alleviating diabetes (Lee et al., 2013; Li et al., 2019), regulating lactase activity (Hajare and Bekele, 2017), and producing vitamins (Greppi et al., 2017; Li et al., 2017). As a symbiotic organism, it may play an important role in the host health maintenance and disease control. Oral *Lactobacillus* to support physiological and physical functions, thereby reducing the risk of disease or shortening the duration or severity of disease, has become an important means of treatment or prevention of disease. There are numerous preclinical and clinical studies confirming the gastrointestinal benefits of *Lactobacillus* in healthy individuals and in a wide range of both minor and serious health conditions (de Simone, 2019). As a result, sales of probiotic-based health products, including pharmaceuticals, dietary supplements, and functional foods, are growing rapidly. Meanwhile, *Lactobacillus* has become the most common probiotic preparation in the market, with good development prospects (Mazzantini et al., 2021).

Adverse environments such as high or low temperature, low pH, bile salts, oxygen, or limited nutrition can cause stress inducing that affect LAB survival during processing and storage, as well as survival, proliferation, and function in the gastrointestinal tract (GIT; Bron and Kleerebezem, 2011; Mills et al., 2011). Among them, oxidative stress is critical important, which can greatly affect the survival ability of LAB. High oxygen levels can lead to the formation and accumulation of reactive oxygen species (ROS), including superoxide anions (O_2_^−^), hydrogen peroxide (H_2_O_2_), and highly reactive hydroxyl radicals (HO•), and cause cell damage, affecting their physiological functions (Amaretti et al., 2013). Therefore, improving the oxidative stress ability of LAB cells is crucial to ensure high bacterial activity in storage and gastrointestinal tract (Feng and Wang, 2020).

In addition, a great quantity of researches reported that some LAB strains have good antioxidant potential in recent years and can be used as a high-quality natural antioxidant (Mishra et al., 2015). Ingestion of these probiotics and their fermented products can help remove ROS from the host gut, reduce the risk of oxidative damage to host cells and the incidence of chronic diseases (Amaretti et al., 2013). For instance, pretreatment with *L. plantarum* ZLP001 could protected IPEC-J2 cells against H_2_O_2_-induced oxidative damage as indicated by cell viability assays and significantly alleviated apoptosis elicited by H_2_O_2_ (Wang et al., 2021). Therefore, the antioxidant capacity of LAB is gradually attracting people’s attention. Various methodologies have been used to assess the antioxidative properties of LAB *in vitro* and *in vivo*. The common assays *in vitro* were based on determining O_2_ tolerance (Li et al., 2010), resistance to H_2_O_2_ (Bchmeier et al., 1997), the reducing power (Tang et al., 2017), radical production and scavenging capacity including ABTS scavenging assay, DPPH scavenging assay (Mu et al., 2019), superoxide radical scavenging assay (Balcazar et al., 2007), hydroxyl radical scavenging assay (Gao et al., 2013), and oxygen radical absorbance capacity (ORAC) assay (Persichetti et al., 2014). The *in vivo* experiments involved establishing animal models, such as the D-galactose-induced aging mouse model (Tang et al., 2016; Lin et al., 2018) and UV-irradiated hairless mouse model (Ishii et al., 2014), and were based on determining physiological and biochemical indexes of experimental animal blood or tissues to evaluate the antioxidants’ AOCs. At present, plenty of LAB strains with antioxidant effects through these experiments was screened out, such as *L. plantarum* 21, *Lactobacillus* sp. SBT-2028, *L. fermentum* ME-3, *L. plantarum* AR113 and *L. casei* KCTC 3260 (Kaizu et al., 1993; Lee et al., 2005; Ou et al., 2009; Achuthan et al., 2012; Lin et al., 2020). However, the antioxidant mechanisms of probiotic LAB are complex, and different strains use different mechanisms. It has been suggested that LAB may play antioxidant roles through scavenging ROS, chelating metals, increasing antioxidant enzymes levels, and modulating the microbiota (Feng and Wang, 2020). It is necessary to study the antioxidant properties of different strains for systematically revealing the antioxidant mechanism of LAB.

*L. plantarum* strain GXL94 with potential antioxidant capacity was isolated by hydrogen peroxide tolerance assay of 113 LAB strains obtained from fermented food. In the present study, we report the *in vitro* evaluation of probiotic properties and antioxidant activity of this isolate. The purpose of this research was to screen the functional LAB with good quality and lay a foundation for rational development and utilization of LAB.

## Materials and methods

### Bacterial strains and culture condition

Twenty-four samples of traditional fermented chili were collected from the domestic producers in Guangxi province in China. These samples were transferred to the laboratory and stored at refrigerator 4°C. The samples were enriched by adding 1% volume to 50 ml of sterile de Man Rogosa Sharpe (MRS) broth and incubated at 37°C for 48 h. 100 μl of each sample were spread over solidified MRS medium and incubated 24 h at 37°C. Then, five single colonies with distinct morphology were picked from each sample and subjected to initial morphological and biochemical tests. One hundred and thirteen cultures were obtained in total and they were identified using the 16S rDNA sequences. The genomic DNA of the strain was extracted and then performed PCR amplification with universal primers. Finally, the PCR product was recovered and sequenced. The sequencing results came from the Changsha Qingke Biological Co., Ltd. Sequencing results were compared on the NCBI website for homology comparison. All strains were stored in de Man, Rogosa, and Sharpe (MRS) broth with 40% glycerol at −80°C and isolated on MRS agar plates. Before all experiment, they were transferred three times for high activity in MRS broth after activated at 37°C for 16 h.

### Tolerance analysis to hydrogen peroxide of LAB

The previously reported method was used with some modifications (Bchmeier et al., 1997). The overnight cultures were adjusted to the same bacterial concentration and inoculated at 1% v/v into MRS broth containing 13 mM H_2_O_2_. After incubation at 37°C for 2 h, the cultures were plated onto MRS agar and incubated at 37°C for 48 h. The bacterial colonies were counted.

Three LAB strains were harvested by centrifugation after cultured for 18 h. GXL94, GXL50, SCL43 cells were resuspended and cultivated at 37°C for 2 h in fresh sterilized MRS broth containing different concentrations of H_2_O_2_ (0, 2, 4, 6, 8, 10, 12, 14, 16, 18, 20, and 22 mM) after adjusted OD_600_ to 0.80. Plate count method was used to count the LAB cells immediately after cultivated. The survival rate was calculated according to Guo et al. (2009).

### Bioscreen assay

To analyze the biomass proliferation, pure cultures were inoculated (3%, v/v) into MRS broth supplemented with different concentrations of H_2_O_2_ (1.0, 2, 3, 4, 5, and 6 mM) and incubated at 37°C for 60 h. The control medium contained no H_2_O_2_. Every 1 h, the plate was shaken briefly, and the OD_600_ was measured by microplate reader.

### Expression levels of genes involved in antioxidation

*Lactobacillus plantarum* GXL94 activated was inoculated (3%, v/v) in MRS broth containing different concentrations of H_2_O_2_ (0, 2.0, and 3.0 mM), and the LAB cells were harvested at different phase (lag phase, mid-logarithmic phase, primary stationary phase or middle-stationary phase) for RNA extraction. The total RNA of LAB cells were extracted and reversed into cDNA. These cDNAs were used as the template for determined antioxidant related gene expression level. The primers used were according to previous reported (Tang et al., 2017) and 16S rRNA was used as a house-keeping gene. The real-time PCR data was analyzed by using the 2^−ΔΔCT^ assay and expressed as n-fold change relative to the experimental control (0 mM H_2_O_2_).

### Detection of antioxidation in fermented supernatant, intact cells, and cell-free extract of GXL94

GXL94 was cultured in MRS broth at 37°C and the intact cells and fermented supernatant were harvested by centrifugation (6,000 g for 10 min at 4°C) after incubation for 18 h. The cells were washed three times with isotonic saline (0.9%) and resuspended in an equal volume of isotonic saline. The cell pellet was adjusted to 1 × 10^10^ CFU/ml. Cell-free extracts were obtained from cell suspensions containing 1 × 10^10^ CFU/ml that were subjected to ultrasonic disruption (ten 5-s strokes at 0°C with 5-s intervals, 10 min; Scientz-IID, China) and centrifuged (6,000 g for 10 min at 4°C) to remove the cell debris. *In vitro* antioxidative activities of these bacterial samples were evaluated in terms of the scavenging rates of 1,1-diphenyl-2-picryl-hydrazyl (DPPH•), superoxide anion, 2,2′-azino-bis(3-ethylbenzothiazoline-6-sulfonic acid; ABTS•), hydroxyl radicals and superoxide anion radical as described (Tang et al., 2017; Mu et al., 2019). The total reducing power assay was performed according to a reported method (Tang et al., 2017), and ascorbic acid was used as the standard to determine the reducing activity.

### Growth of LAB under acid, bile salts, and NaCl

The LAB strains were inoculated (2%, v/v) into MRS broth and the cells were harvested by centrifugation after cultured for 18 h. Then the collected cells were treated with different initial pH (2.5, 3.0, 4.0, 5.0), various bile concentration (0.3%–2%, w/v) or various sodium chloride (NaCl) concentration (2.0%–8.0%, w/v) for 3 h, the survival rates were measured after treatments. The viable count before incubation was measured as control (CK).

### Survival to simulated gastrointestinal tract transit

Survival to simulated gastrointestinal tract (GIT) conditions was evaluated according to the previous study (Lin et al., 2018). Intact cells (OD_600_ = 1.0) were resuspended in simulated gastric juice (SGJ:25 mM NaCl, 7 mM KCl, 45 mM NaHCO_3_, 3 g/l pepsin, pH 3.0) and incubated at 37°C for 3 h at 200 rpm. SGJ-treated cells were harvested by centrifugation (10,000 ×g for 5 min at 4°C), resuspended in equal volume of simulated pancreatic juice (0.15 g/100 ml bile salt, 1 g/L trypsin, pH 8.0), and incubated at 37°C for 3 h at 200 rpm. The cells incubated in normal saline were used as controls. The survivors were counted by pour plating on MRS agar (incubation at 37°C for 48 h).

### Auto-aggregation assay

The auto-aggregation ability was performed according to Lin et al. (2020). The stain of LAB cultured for 18 h were harvested by centrifugation (10,000 rpm, 10 min at 4°C). The sediment was re-suspended in sterile normal saline and adjusted to 10^9^ CFU/ml after washed thrice with sterile normal saline. Next, the suspensions of LAB were incubated at 37°C for 5 h. The optical density at 600 nm of upper layer suspension was measured at 0, 1, and 5 h by a UV spectrophotometer without hanging the microbial suspension. Auto-aggregation ability was calculated as follows: Auto-aggregation ability (%) = (1 − A_t_/A_0_) × 100%.

Where A_t_ is OD_600_ at 1, 3, or 5 h, and A_0_ is OD_600_ at 0 h.

### Co-aggregation assay

The co-aggregation ability was performed according to Lin et al. (2020) with modifications. The strain of LAB and *E. coli* O157: H7 were harvested as described above. The LAB and *E. coli* O157:H7 cells were adjusted to 0.25 and 0.60 at OD_600_ after washed three times with normal saline, respectively. Equal volumes (5 ml) of different LAB and *E. coli* cells suspension were mixed together by vortexing for 10 s. The absorbance (A_mix_) of upper layer of the suspension at 600 nm was measured after incubation at 37°C for 5 h. The percentage of co-aggregation was calculated as follows:

Co-aggregation ability (%) = [((A_1_ + A_2_)/2−A_mix_)/ (A_1_ + A_2_)/2] ˟100%.

Where A_1_ and A_2_ represent absorbance of the two strains at 0 h, respectively. A_mix_ represent absorbance of the mixture after 5 h of incubation.

### Cell surface hydrophobicity assay

The cell surface hydrophobicity of LAB was determined according to previous report with some modifications. The method for preparing the cell suspensions was the same as that in auto-aggregation assay. The mixture of LAB cells and xylene in equal volumes (2 ml) was incubated at room temperature (25°C) for 30 min after mixed by vortexing for 3 min. The adherence of LAB to hydrocarbons was calculated as follows:

Cell surface hydrophobicity (%) = [(A_0_-A_t_)/A_0_] × 100%.

Where A_0_ is OD_600_ before treatment with xylene, and A_t_ is the OD_600_ of the aqueous phase after treatment with xylene.

### Antibiotic susceptibility

Antibiotic susceptibility of LAB strains was evaluated using the disk diffusion method according to the Clinical and Laboratory Standards Institute (CLSI) guidelines (Ahire et al., 2021). Susceptibility to the following 10 antibiotics was measured: 5 mcg of ciprofloxacin, and rifampicin; 10 mcg of ampicillin, gentamicin, and penicillin G; 15 mcg of erythromycin; 30 mcg of tetracycline, chloramphenicol, vancomycin, and kanamycin. LAB strains (1 × 10^8^ CFU/ml) were spread on MRS agar plate, and antibiotic disks were placed on these plates under sterile conditions. After incubation at 37°C for overnight, the diameter of clear zones surrounding the disks was measured in millimeters (mm).

### Hemolytic activity

Hemolytic activity was performed as described by Ahire et al. (2021) with some modifications. Overnight grown culture of *L. plantarum* GXL94 was streaked on 7% defibrinated sheep blood agar plates and incubated at 37°C for 48 h. After incubation, the plates were observed for α-hemolysis (dark and greenish zones), β-hemolysis (lightened-yellow or transparent zones), and γ-hemolysis (no change or no zones).

### Statistical analysis

The results were expressed as mean ± standard error. All experiments were carried out in triplicate. The procedure univariate in SAS was used to analyze data on relative expression value of each gene between different treatments. One-way analysis of variance (ANOVA) and Student’s *t* test were used to test the significant differences (*p* < 0.01 and *p* < 0.001) of means.

## Results

### *Lactobacillus plantarum* GXL94 was resistant to hydrogen peroxide

In this study, three cultures exhibited a moderate-to-strong resistance to H_2_O_2_. *L. plantarum* GXL94 and GXL50 was the most resistant strain against H_2_O_2_ with more than 100 CFU in the plate. *Lactobacillus pentosus* SCL43 was less tolerance than GXL94 and GXL50, however it also showed a certain tolerance to H_2_O_2_ (Table 1). Thus, these three strains were chosen to examine the tolerance to different concentration of H_2_O_2_.

**Table 1:** Resistance of LAB strains to hydrogen peroxide.

CFU/100 (μl)	Total	LAB strains number
<10	109	
10~49	1	GXL97
50~100	1	SCL43
>100	2	GXL50, GXL94

The tolerate ability of GXL94, GXL50 and SCL43 to different concentrations of H_2_O_2_ were presented in Table 2. The survival rate of three strains were decreased with the concentration of H_2_O_2_ increasing. The survival rates were over 50% at 14 mM H_2_O_2_ for GXL94 or GXL50 and at 12 mM H_2_O_2_ for SCL43. When concentration of H_2_O_2_ up to 22 mM, there was still a survival rate of 19.64% for GXL94. However, at the same concentration of H_2_O_2_, there was no viable LAB colonies for GXL50 and SCL43 after cultivated for 2 h. This result indicated that strain GXL94 possessed a stronger antioxidant property.

**Table 2:** Survival rates (%) of LAB in different concentrations of hydrogen peroxide.

Concentrations of H_2_O_2_	GXL94		GXL50		SCL43	
	CFU/ml	Survived rate (%)	CFU/ml	Survived rate (%)	CFU/ml	Survived rate (%)
0 mM	(5.43 ± 0.21) × 10^8^	100%	(5.5 ± 0.26) × 10^8^	100%	(1.36 ± 0.01) × 10^9^	100%
2 mM	(5.33 ± 0.58) × 10^8^	99.91%	(5 ± 0.1) × 10^8^	99.53%	(1.06 ± 0.06) × 10^9^	98.81%
4 mM	(4.93 ± 0.8) × 10^8^	99.52%	(5.4 ± 0.26) × 10^8^	99.91%	(7.1 ± 0.3) × 10^8^	96.91%
6 mM	(4.29 ± 0.79) × 10^8^	98.83%	(4.03 ± 0.86) × 10^8^	98.45%	(1.26 ± 0.17) × 10^8^	88.69%
8 mM	(1.65 ± 0.15) × 10^8^	94.08%	(2.53 ± 0.51) × 10^8^	96.13%	(8.81 ± 0.82) × 10^7^	86.99%
10 mM	(1.12 ± 0.19) × 10^7^	80.72%	(9.3 ± 0.36) × 10^6^	79.73%	(6.18 ± 1.7) × 10^7^	85.30%
12 mM	(1.73 ± 0.15) × 10^6^	71.40%	(1.81 ± 0.29) × 10^6^	71.60%	(2.75 ± 0.05) × 10^6^	70.50%
14 mM	(4.13 ± 0.55) × 10^4^	52.85%	(3.64 ± 0.84) × 10^4^	52.18%	(1.42 ± 0.01) × 10^4^	45.47%
16 mM	(1.19 ± 0.07) × 10^4^	46.66%	(5.51 ± 1.32) × 10^3^	42.80%	(3.63 ± 0.24) × 10^3^	38.98%
18 mM	(1.1 ± 0.17) × 10^3^	34.82%	(3.53 ± 0.62) × 10^2^	29.15%	(4.74 ± 0.04) × 10^2^	29.29%
20 mM	(1.45 ± 0.24) × 10^2^	24.74%	(2.73 ± 0.64) × 10^1^	16.44%	(4.45 ± 0.45) × 10^1^	18.05%
22 mM	(5.2 ± 0.8) × 10^1^	19.64%	0	0.00%	0	0.00%

### Growth of GXL94 at different concentrations of H_2_O_2_

**Figure 1:**
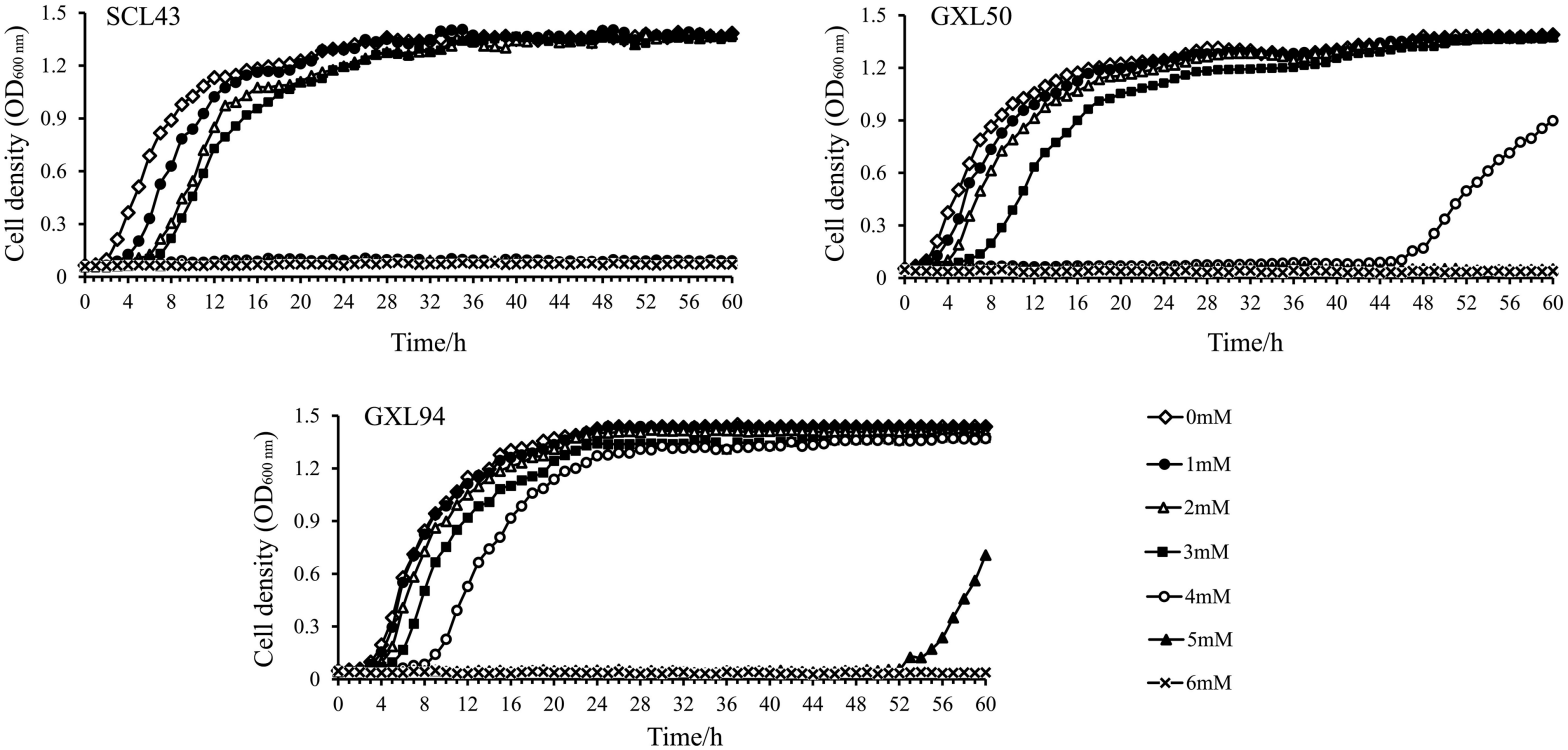
Growth density of *Lactobacillus pentosus* SCL43, *Lactobacillus plantarum* GXL50 and GXL94 under oxidative stress. Experiments were conducted in MRS containing different concentrations of H_2_O_2_ (1, 2, 3, 4, 5, and 6 mM).

As mentioned above, H_2_O_2_ could induced oxidative stress of LAB. The growth of SCL43, GXL50 and GXL94 was inhibited by H_2_O_2_ as shown in Figure 1. The addition of H_2_O_2_ inhibited the growth and the lag phase was obviously elongated with the concentration of H_2_O_2_ increasing, which indicating that the presence of H_2_O_2_ cause bacterial oxidative damage. The further results showed that the growth inhibition of GXL94 in the range from 0 to 5 mM, and the growth was ceased as concentration amount to 6 mM H_2_O_2_. These results showed that GXL94 could survived the challenge of H_2_O_2_ up to 5 mM, higher than SCL43, GXL50 and other strains reported before.

### Analysis of the transcript levels of the antioxidant-related genes in strains exposed to H_2_O_2_

**Figure 2:**
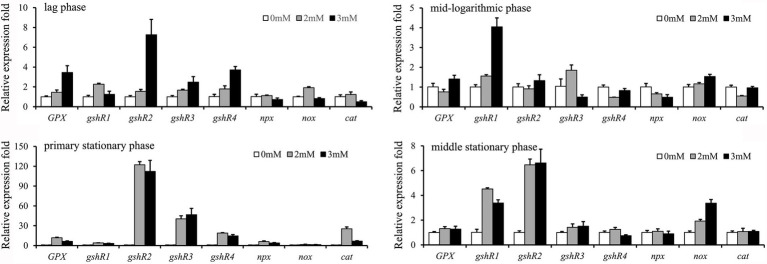
Effects of the addition of hydrogen peroxide on the expression of 8 antioxidant-related genes in *Lactobacillus plantarum* GXL94. The graphs show the relative mRNA levels of 8 genes of cells grown in MRS medium with different concentrations of hydrogen peroxide at the lag phase, mid-logarithmic phase, primary stationary phase and middle stationary phase.

Relative expression levels of eight antioxidant-related genes were detected when exposed to different concentrations of H_2_O_2_ (Figure 2). The result showed that the expression levels of four genes including *GPX*, *gshR2*, *gshR3*, and *gshR4* in GXL94 at lag phase were increased with increasing of H_2_O_2_ concentration, and shown positive correlation, however *gshR1*, *npx*, *cat*, and *nox* gene with no apparent correlation between H_2_O_2_ and expression levels. The expression levels of *gshR1* and *nox* at the mid-logarithmic phase were positive correlation with the increasing of H_2_O_2_ concentration, while the genes expression levels of *npx* was decreased with the increasing H_2_O_2_ concentration, moreover there are no apparent correlation between H_2_O_2_ and mRNA level of other six genes. At primary stationary phase, all of eight antioxidant-related genes were significantly upregulated with H_2_O_2_ treatment, but they were no apparent correlation. At middle stationary phase, the expression level of *gshR1*, *gshR3* and *nox* were significantly induced, while only the gene *nox* expression level showed an apparent correlation with H_2_O_2_ concentration.

Among the induced transcription of eight genes related to antioxidation at primary stationary phase, expression of *GPX* was increased by 11.73-fold and 6.26-fold under 2.0 mM and 3.0 mM H_2_O_2_ challenge, respectively. Expression level of *gshR1* was increased by 3.99-fold and 3.22-fold under 2.0 mM and 3.0 mM H_2_O_2_ challenge, respectively. The expression level of *gshR2* was increased by 122.28-fold and 112.22-fold treated with 2.0 mM and 3.0 mM H_2_O_2_, respectively. In addition, *gshR3* and *gshR4* mRNA expression was elevated by 40.32-fold/18.86-fold and 46.71-fold/14.67-fold in response to the treatment with 2.0 mM and 3.0 mM H_2_O_2._ The cumulative values of the eight genes’ expression at primary stationary phase were largest of the four cultivation stages, which indicate that GXL94 exhibited higher antioxidant activity in the primary stationary, the GSH system was supposed to have played a major role in antioxidant activity.

### Antioxidant activity *in vitro*

The antioxidant mechanisms of probiotic LAB are complex, and different strains use different mechanisms. Various approaches have to be combined in order to identify and characterize the antioxidant activity of LAB. In this study, five commonly used antioxidant indexes of GXL94 *in vitro* were analyzed (Table 3).

**Table 3:** Antioxidant activity of different samples of *Lactobacillus plantarum* GXL94.

Subjects	Fermented supernatant	Intact cell	Cell-free extract
Reducing activity (μmol/L ascorbic acid equivalent)	-	185.66 ± 5.94	262.88 ± 12.30
Scavenging of DPPH∙(%)	95.98 ± 1.59	83.82 ± 0.71	38.84 ± 1.93
Scavenging of ABTS (%)	94.63 ± 0.54	89.47 ± 0.36	89.61 ± 0.31
Scavenging of ∙OH (%)	88.47 ± 0.32	61.43 ± 0.82	86.01 ± 0.28
Scavenging of ∙O_2_^−^ (%)	34.41 ± 1.4	10.53 ± 3.1	14.47 ± 1.73

Reducing activity of different samples of the fermentate of GXL94 was measured and expressed as an equivalent amount of ascorbic acid. The cell-free extract exhibited the stronger reducing activity (262.88 ± 12.30 μmol/L ascorbic acid equivalent) than the intact cells (185.66 ± 5.94 μmol/L ascorbic acid equivalent).

Scavenging of DPPH free radical is attributed to the hydrogen donating ability of antioxidants and is routinely used for antioxidant assay. Experiment result indicated that the fermented supernatant (95.98% ± 1.59%) and intact cells (83.82% ± 0.71%) of *L. plantarum* GXL94 exhibited more stronger DPPH scavenging ability compared to the cell-free extract (38.84% ± 1.93%; *p* < 0.05).

ABTS radical cation-scavenging activity was also an important index for antioxidant capacity analysis. In this study, ABTS scavenging ability was evaluated, and the result showed that the intact cells and cell-free extract exhibited a high scavenging activity, with ratios at 89.47% ± 0.36% and 89.61% ± 0.31%, respectively (Table 2). However, which were still slightly weaker than fermented supernatant (94.63% ± 0.54%).

Fenton reaction was used to measure the scavenging activities of hydroxyl radicals. The fermented supernatant (88.47% ± 0.32%) and cell-free extract (86.01% ± 0.28%) demonstrated a significantly higher scavenging capacity, compared to that of intact cells (61.43% ± 0.82%)(*p* < 0.05).

Through the improved pyrogallol autoxidation method, it was found that all of samples exhibited •O_2_^−^-scavenging activity, and the clearance rate of fermentation supernatant was highest, reached 34.41% ± 1.4%. In conclusion, *L. plantarum* GXL94 has a great antioxidant activity *in vitro*, and has promising antioxidant potentials in therapeutic benefits for human health.

### Tolerances to acid, bile salt, and NaCl

**Figure 3:**
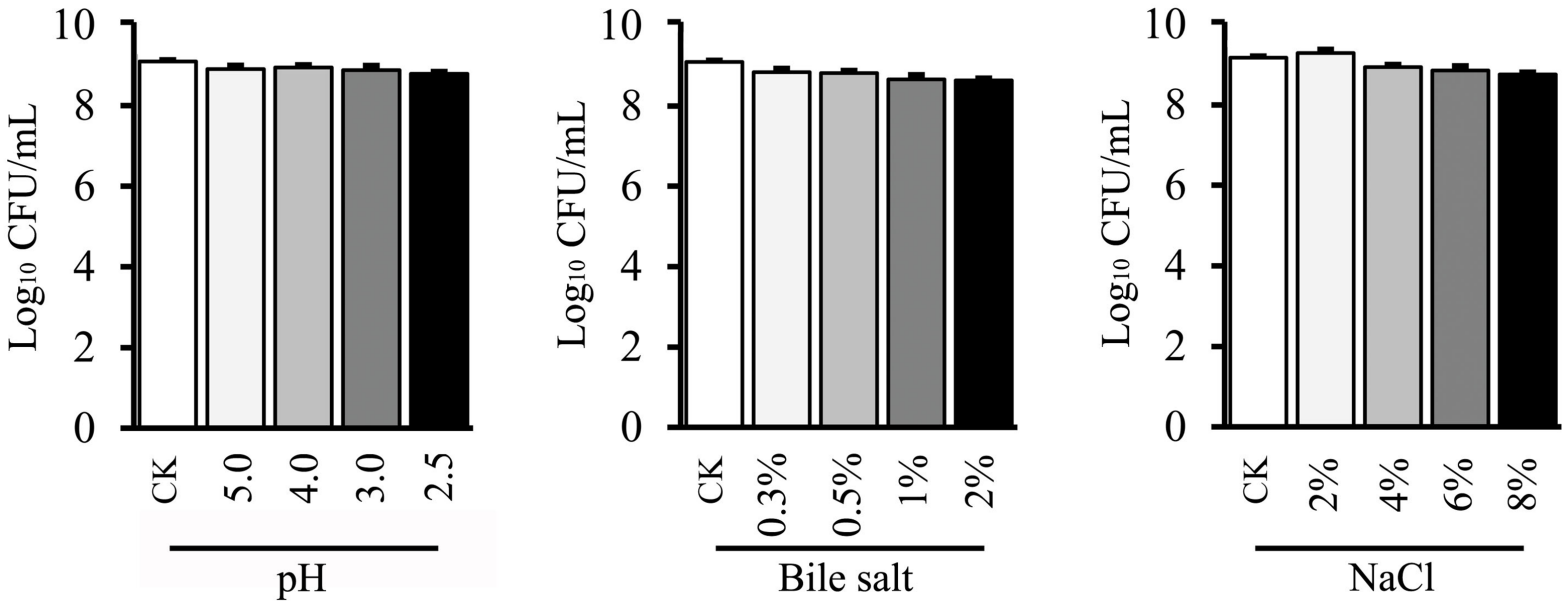
Effect of low pH, bile salt, and NaCl on viable count of *Lactobacillus plantarum* GXL94.

Tolerances to low acid and high bile stresses are significant properties for any potential probiotic bacteria. The abilities of LAB to tolerate the pH and bile salt were presented in Figure 3. The gradual decreased survival of *L. plantarum* GXL94 (pH 5.0 98.07%; pH 4.0 98.04%; pH 3.0 97.8%; pH 2.5 96.7%) was noticed when the cells were incubated at different pHs as compared with control (Figure 3A). Moreover, a similar decreased pattern (97.34%; 97.09%; 95.37%; 95.09%) was observed with the concentration of bile salt treatment increases. Besides this, the viable cells of GXL94 was increased under the condition of 2% NaCl due to proliferation, but decreased significantly as osmotic pressure increasing (4%: 97.4%; 6%: 96.5%; 8%: 95.4%) as compared with the control (Figure 3). These results indicated that GXL94 has good tolerance to low acid and high concentration of bile salts and NaCl treatment.

### Survival to simulated gastrointestinal tract transit

**Figure 4:**
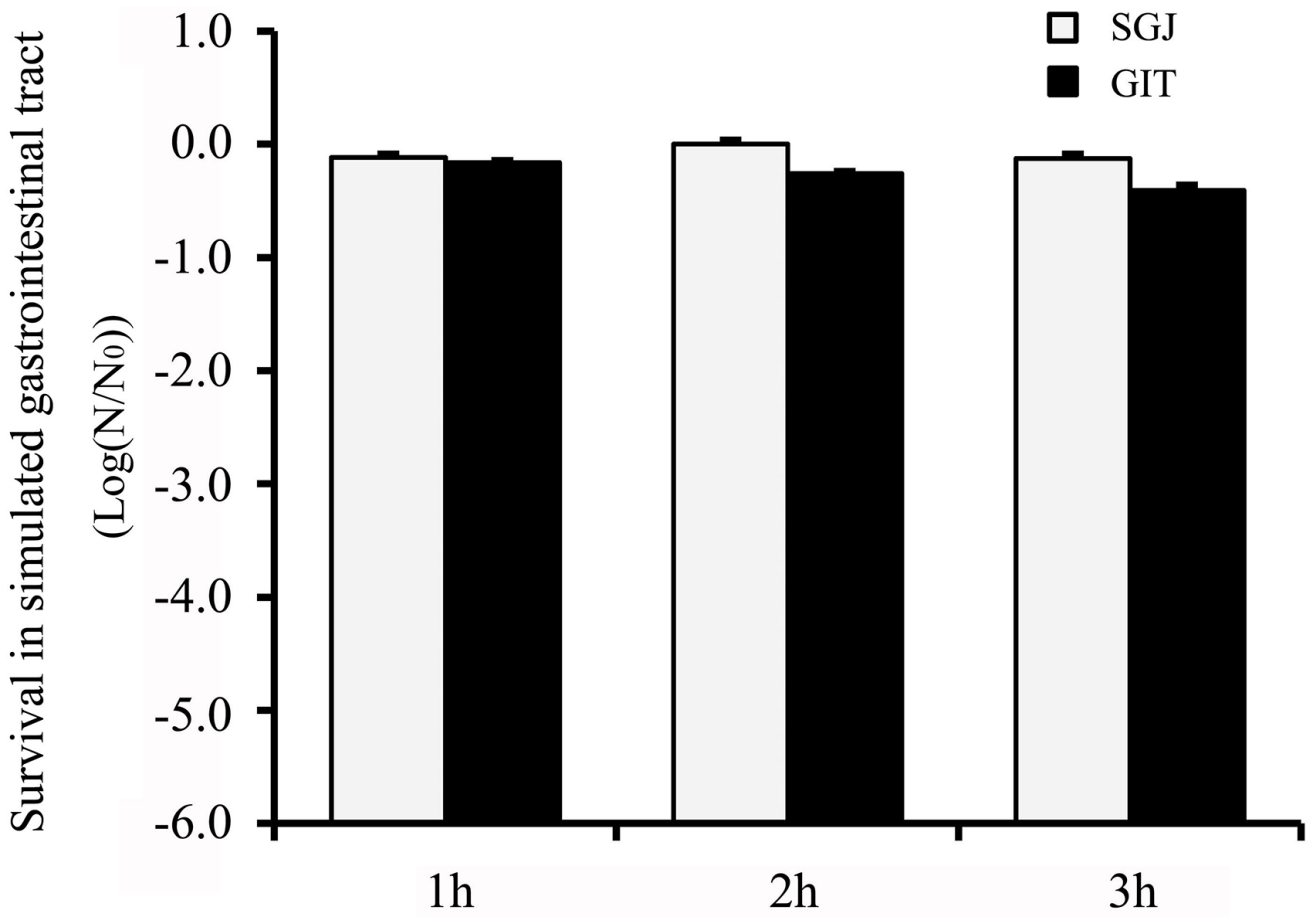
Tolerance of simulated gastrointestinal tract (GIT) transit of the cells of *Lactobacillus plantarum* GXL94. Survival was expressed as reduction of log (N/N0) cycles, where N_0_ and N are the number of viable cells, respectively, before and after exposure to stress; standard error bars are shown.

The isolates were further subjected to survival in simulated gastrointestinal tract (GIT) conditions, and the survivors (log N/N_0_) to SGJ and simulated GIT juices are shown in Figure 4. GXL94 showed good survival percentage in both conditions for 3 h, with the survival rate above 99% in 3 h in SGJ and above 95% in 3 h in GIT juices. Comparatively, GXL94 exhibited the strongest resistance to gastric treatment, but the lowest survival to pancreatic juice. Thus, the intact cells of GXL94 could live through simulated GIT in colonizing the GIT of the host.

### Adhesion potential

Bacterial adhesion to hydrocarbon was evaluated, and as shown in Table 4, the percentage hydrophobicity for GXL94 cells showed 33.01 ± 3.65% for xylene. The co-aggregation ability of GXL94 with the pathogen *E. coli* O157:H7 was only 30.87% ± 0.21% after incubation at 37°C for 5 h. In addition, GXL94 showed an auto-aggregation percentage of 10.63%, 29.48%, and 50.45% after 1, 3, and 5 h of incubation, respectively.

**Table 4:** Auto-aggregation, Co-aggregation and cell surface hydrophobicity of the LAB.

Strains	Auto-aggregation (%)			Co-aggregation (%)	Cell surface hydrophobicity (%)
	1 h	3 h	5 h		
GXL94	10.63 ± 2.36	29.48 ± 0.34	50.45 ± 4.16	30.87 ± 0.21	33.01 ± 3.65

### Antibiotic susceptibility and hemolytic activity

Tolerance of the strain GXL94 to 10 kinds of antibiotics was shown in Table 5. As is known to all, the susceptibility and resistance of LAB to various antibiotics are variable depending on the species (Li et al., 2020). *L. plantarum* GXL94 showed sensitivity toward antibiotics belonging to the class of β lactam, macrolactams, nitrobenzenes (Table 5). Meanwhile, GXL94 was resistant to quinolones, aminocyclitol glycosides, polyketide, glycopeptides, aminoglycosides class of tested antibiotics (Table 5). Besides this, GXL94 also was found intermediary sensitive to macrolides. Our results indicated that this LAB strain exhibited a good antibiotic susceptibility.

**Table 5:** Antibiotic susceptibility of *Lactobacillus plantarum* GXL94.

Class of antibiotic	Antibiotic	Concentration	Zone of inhibition in millimeter (mm)	Breakpoints as per CLSI and EUCAST
β lactam	Ampicillin	10 mcg	29.2	S
	Penicillin G	10 mcg	30.35	S
Quinolones	Ciprofloxacin	5 mcg	8.68	R
Aminocyclitol glycosides	Gentamicin	10 mcg	6	R
Polyketide	Tetracycline	30 mcg	10.6	I
Macrolactams	Rifampicin	5 mcg	26.6	S
Nitrobenzenes	Chloramphenicol	30 mcg	27.13	S
Glycopeptides	Vancomycin	30 mcg	6	R
Macrolides	Erythromycin	15 mcg	17.55	I
Aminoglycosides	Kanamycin	30 mcg	6	R

In addition, GXL94 showed no zone of hemolysis when streaked onto sheep blood agar plates, which was indicative of γ-hemolytic activity (non-hemolytic). And these result further confirmed the biosafety of this strain.

## Discussion

H_2_O_2_ is a weak oxidant, but can permeate the cell membrane and form more active ROS, such as hydroxyl radicals, *via* the Fenton reaction, auto-oxidation and other reactions thereby causing oxidative damage subsequently (Mishra et al., 2015). It is worth mentioning that lactic acid bacteria are regarded as catalase-negative microorganisms used to produce fermented foods (Mokoena et al., 2021). In recent years, a plenty of LAB strains resistant to hydrogen peroxide have been reported. For instance, *L. plantarum* KCC-24 showed moderate resistance to H_2_O_2_ as which can grow normally under the condition of 1.5 mM hydrogen peroxide (Vijayakumara et al., 2015). *L. plantarum* Y44 had a higher survival rate (>55%) when exposed to 1.0 mM hydrogen peroxide (Mu et al., 2018). Moreover, the latest research confirms that *L. plantarum* AR113 could tolerance against 8.0 mM H_2_O_2_, and it turned into exponential phase at 48 h under 3.5 mM H_2_O_2_ (Lin et al., 2020)_._ In this study, we confirmed that *L. plantarum* GXL94 has a stronger hydrogen peroxide resistance than these antioxidant strains mentioned above, as it can tolerate up to 22 mM hydrogen peroxide concentration.

Most antioxidant evaluation methods based on reactive species can be applied to probiotics, including intact cells, cell-free extracts and cell lysates or their metabolic products to evaluate their antioxidant capacity *in vitro* (Lin and Chang, 2000; Zolotukhin et al., 2018; Noureen et al., 2019). In this study, we evaluated the antioxidant effects of this three GXL94 samples *in vitro* respectively, and found both fermented supernatant and intact cells have a good antioxidant potential capacity. Previous research results show that antioxidant activity of LAB strains could be attributed to their production of cell-surface active compounds including proteins, lipoteichoic acid or extracellular polysaccharides (Li et al., 2012; Polak-Berecka et al., 2013), carotenoids (Kim et al., 2019), ferulic acid (Bhathena et al., 2008), or histamine (Azuma et al., 2001). Therefore, whether GXL94 can produce antioxidant molecules to improve its antioxidant performance remains to be further verified.

Antioxidant enzymes including SOD and catalase, thioredoxin, NADH oxidase-NADH peroxidase system, and glutathione system are regarded as three main enzymatic defense mechanisms against oxidative stress in LAB (Serrano et al., 2007; Feng and Wang, 2020). Catalase plays an important role in reducing oxidative stress by decomposing H_2_O_2_. LAB have been classified for a long time as unable to produce catalase because of the lack of heme (Ricciardi et al., 2018). However, several researches in recent years have demonstrated that at least some LAB strains are able to synthesis a heme-containing catalase, such *L. plantarum* CNRZ 1228, *L. sakei* YSI8, *L. brevis* CGMCC1306 (Abriouel et al., 2004; An et al., 2010; Lyu et al., 2016). The presence of this gene can significantly improve LAB antioxidant activity. We also cloned heme-containing catalase gene (*cat*) in strain GXL94, and the transcription of this gene was elevated by 25.3-fold and 6.8, when treated with 2 mM and 3 mM H_2_O_2_ at primary stationary phase, respectively. Coupled NADH oxidase—NADH peroxidase system was a common oxidative stress resistance mechanism found in LAB. In these coupled reactions, intracellular oxygen is first used to oxidize NADH into NAD^+^ by NADH oxidase, thereby releasing H_2_O_2_. Subsequently, H_2_O_2_ is reduced to H_2_O by NADH peroxidase (Miyoshi et al., 2003). In this study, we found that two key genes (*npx* and *nox*) of these system were present in GXL94, and the *npx* gene was significantly up-regulated 3.8–6.1-fold at primary stationary phase stage in the presence of hydrogen peroxide. To some extent, the antioxidant capacity of bacteria is related to the biosynthesis or accumulation of glutathione. Bacteria with fully operational glutathione system can directly detoxify or eliminate H_2_O_2_ and lipid peroxyl radicals, and thus have defense against H_2_O_2_ accumulation (Kullisaar et al., 2010). Previously, it was thought that gram-positive bacteria cannot synthesize GSH and thus do not have the GSH-glutaredoxin system. However, later studies revealed that some LAB, such as and *Lactobacillus fermentum* E3 and E18, naturally synthesize GSH at a high level (Kullisaar et al., 2002). Whereafter, a fully functional GSH system comprising both GSH peroxidase and GSH reductase in *L. fermentum* strain ME-3 was first time discovered by Kullisaar and colleagues (Kullisaar et al., 2010). We also identified a functional GSH system in GXL94 in present study. It was noteworthy that the transcription of the four *gshR* genes were elevated by 3.22-112-fold when GXL94 was challenged with high concentration of H_2_O_2_ (3.0 mM). Moreover, *GPX* function as a H_2_O_2_ receptor and redox transducer, which can directly detoxify or eliminate hydrogen peroxide and peroxyl radicals (Kullisaar et al., 2010). Our results also confirmed that the gene was significantly up-regulated under hydrogen peroxide stress. Therefore, we speculate that the GSH system played a major role to ensure the survival of GXL94 under hydrogen peroxide pressure. In recent years, some researchers have used omics studies to reveal the molecular mechanism of the antioxidant performance of *L. plantarum.* The results showed that various metabolic pathways such as base excision repair system, recombinational DNA repair pathway, pyruvate metabolism, carbon metabolism, trichloroacetic acid cycle, amino acid metabolism, and microbial metabolism were closely related to these antioxidant properties (Zhai et al., 2020; Tian et al., 2022). In future studies, we can use these methods to further explore the molecular mechanism of GXL94 antioxidant properties.

Lactic acid bacteria mainly play a probiotic role in the intestinal tract. After oral ingestion, it needs to experience adverse survival factors in the gastrointestinal tract, such as low pH of gastric acid, bile salts, digestive enzymes, etc. Tolerance to gastrointestinal tract is an important indicator for screening and evaluating effective probiotics. *Lactobacillus paracasei* FM-LP-4 could survive pH 2.5 for 3 h, but the log CFU/ml was greatly reduced from initial 9 to 7 after 3 h and also showed tolerance to 0.5% bile (Wang et al., 2016). Adesulu-Dahunsi et al. (2018) reported the survival rate of *L. plantarum* strain OF101 was 98.4% and 96.9% at pH 2.5% and 0.3% bile, respectively. The results of probiotic characteristic tests showed that MA2 could survive at pH 2.5% and 0.3% bile salt for 3 h and maintained a survival of 70% (Tang et al., 2017). In addition, commercial probiotic *L. casei* Zhang showed survival of 82.7%/97.4% in simulated gastric juice or intestinal juice (Guo et al., 2009). In another study, the survival percentage of *L. plantarum* AR113 in simulated fluids were only 86.7% (Lin et al., 2018). In view of these reports, our isolate GXL94 were found to show better performance in terms of survival in GIT.

The adhesion ability of lactic acid bacteria to host intestinal cells is another important indicator to evaluate its probiotic property. Auto-aggregation, co-aggregation and adhesion to solvents are the measures to estimate bacterial ability to colonize the intestinal wall. In present study, the auto-aggregation percentage observed with the strain GXL94 (50.45%, 5 h) was higher as compared with the *L. plantarum* AR113 (30.1%), 1KMT (45.63%), 4 BC (39.56%; Kaktcham et al., 2018; Lin et al., 2020). The adhesion of GXL94 to xylene was comparatively higher than *L. plantarum* strains UBLP40 (20.4%) and 1KMT (23.71%) reported for xylene adhesion in 1 h (Kaktcham et al., 2018; Ahire et al., 2021). These results indicate that our strain are capable of adhering to epithelial cells and mucosal surfaces. Moreover, GXL94 also showed 30.87% co-aggregation with *E. coli*. Co-aggregation of probiotic LAB strains with pathogenic bacteria enables them to form a barrier that may facilitate the colonization of the pathogen in the gastrointestinal tract. Low levels of co-aggregation may play an important role in preventing the formation of biofilms and preventing the persistence of pathogenic species in the GIT (Todorov et al., 2017).

One of the important characteristic of probiotic is the safety for human consumption and the absence of acquired and transferable antibiotic resistance. In this study, we observed that *L. plantarum* GXL94 showed sensitive to ampicillin, penicillin G, tetracycline, rifampicin, chloramphenicol and erythromycin, which was similar to the recent finding of Lin et al. (2020). Some researchers have tested the drug-resistant phenotypes of selected probiotics and found that most LABs are intrinsic resistant to kanamycin, gentamicin, streptomycin, vancomycin and ciprofloxacin (Li et al., 2020). Our result corroborate well with these results. Actually, some probiotic strains with intrinsic antibiotic resistance can help restore the intestinal microbiota after antibiotic treatment (Gueimonde et al., 2013). Some *Lactobacillus* strains could acquired tetracycline resistance through horizontal gene transfer (Campedelli et al., 2019). GXL94 showed moderate tetracycline resistance in this study, therefore, we need to evaluate whether there are mobile genetic elements with horizontal transfer potential on both sides of tetracycline resistance related genes in future studies. In addition, the hemolytic activity was also evaluated as a criteria for selecting probiotic strains. Our result indicated that GXL94 showed γ-hemolytic or no hemolytic activity, and that it is consistent with found in previous studies (Bhushan et al., 2021). Overall, based on the antibiotic susceptibility profile and hemolytic activity *in vitro*, GXL94 could fulfill the primary requirements of a probiotic and make this strain a safe candidate for further validation in future probiotic studies *in vivo*.

Oxidative stress is the cause of various chronic human diseases, such as cancer, diabetes, heart disease, stroke, Alzheimer’s disease, rheumatoid arthritis, cataract, and aging (Mishra et al., 2015). Antioxidants are molecules that interact with free radicals generated in cells and terminate the chain reaction to relieve this oxidative stress before damage is done to the vital molecules. However, in the process, antioxidants are themselves oxidized. Thus, there is a constant need to replenish antioxidant resources as one antioxidant molecule can react with only a single free radical (Halliwell and Gutteridge, 1990). Consequently, it is essential to search for and develop natural nontoxic antioxidants to protect the human body from free radicals and slow the progress of many chronic diseases. LABs have been considered as an emerging source of effective antioxidants in recent years due to their long tradition of safe use and potential intestinal benefits (Nardone et al., 2010). It is a general consensus that LAB supplementation can regulate intestinal microbiota, and this microbiota composition reconstruction has the potential to improve the host redox state (Qiao et al., 2012; Xin et al., 2014). Therefore, it is important to consume dietary supplements or fermented foods that contain high antioxidant activity lactic acid bacteria to delay the development of chronic diseases. In view of its good antioxidant properties and beneficial properties, we believe that GXL94 has a good prospect in the application of functional foods with antioxidant properties.

## Conclusion

In this study, a LAB strain demonstrated good *in vitro* antioxidative effect was screened out through a series of experimental evaluation. *L. plantarum* GXL94 showed good tolerance to high concentration of hydrogen peroxide pressure and strong scavenging capacities to various free radical. After analyzing the eight antioxidant related genes, *GPX* and *gshR* were found to up-regulated greatly after H_2_O_2_ treated, which may provide the evidence for revealing the molecular mechanism of antioxidant activity of GXL94. Besides this, GXL94 also exhibited promising survivability to acid-, bile salt-, osmotic-, gastric juice-, intestinal juice-tolerance tests, and high auto-aggregation. The non-hemolytic activity and antibiotic susceptibility profile of the strain indicated the safety of probiotic for use. Overall, based on the above analysis for antioxidative and probiotic properties, the strain *L. plantarum* GXL94 could be considered as potential antioxidant probiotic for commercial use.

## Data availability statement

The raw data supporting the conclusions of this article will be made available by the authors, without undue reservation.

## Author contributions

YZ, WG, CXi, and YP were responsible for the study design, supervision, and manuscript preparation. CXu and ZZ were responsible for the experiment and analysis. All authors contributed to the article and approved the submitted version.

## Funding

This research was supported by Agricultural Science and Technology Innovation Program of China (CAAS-ASTIP-2022-IBFC), China Agriculture Research System for Bast and Leaf Fiber Crops (no. CARS-16), and Training Program for Excellent Young Innovators of Changsha (kq2106095).

## Conflict of interest

The authors declare that the research was conducted in the absence of any commercial or financial relationships that could be construed as a potential conflict of interest.

## Publisher’s note

All claims expressed in this article are solely those of the authors and do not necessarily represent those of their affiliated organizations, or those of the publisher, the editors and the reviewers. Any product that may be evaluated in this article, or claim that may be made by its manufacturer, is not guaranteed or endorsed by the publisher.
